# PEGylated gas vesicles: a promising novel ultrasound contrast agent for diagnosis and guiding radiofrequency ablation of liver tumor

**DOI:** 10.1186/s12951-025-03377-z

**Published:** 2025-05-14

**Authors:** Kezhi Yu, Yongquan Huang, Yuanyuan Wang, Qunyan Wu, Zihang Wang, Fei Li, Jianri Chen, Maierhaba Yibulayin, Shushan Zhang, Zhongzhen Su, Fei Yan

**Affiliations:** 1 Department of Ultrasound, the Fifth Affiliated Hospital of Sun Yat-sen University, Zhuhai, Guangdong Province China; 2 Key Laboratory of Quantitative Synthetic Biology, Shenzhen Institute of Synthetic Biology, Shenzhen Institutes of Advanced Technology, Chinese Academy of Sciences, 1068 Xueyuan Avenue, Shenzhen University Town, Shenzhen, 518055 Guangdong Province China; 3 Department of Echocardiography, Xinjiang Key Laboratory of Ultrasound Medicine, First Affiliated Hospital of Xinjiang Medical University, Urumqi, Xinjiang Province China; 4 Research Center for Advanced Detection Materials and Medical lmaging Devices, Shenzhen Institutes of Advanced Technology, Chinese Academy of Sciences, Shenzhen, Guangdong Province China; 5 Southern University of Science and Technology, Shenzhen Guangdong Province, China

**Keywords:** Gas vesicles, Ultrasound contrast agents, Biosynthesis, Liver tumor, Diagnosis

## Abstract

Ultrasound contrast agents (UCAs) play an important role in diagnosis and the imaging-guided treatment of liver tumor in clinical settings. However, most commercially available UCAs are micro-sized and fabricated through a chemical synthesis route. Here, we developed a new class of biosynthesized nanoscale contrast agent (PEG-GVs) and comprehensively compared its physicochemical characteristics and imaging performance with commercial Sonovue and Sonazoid. Our results revealed PEG-GVs may produce more stable and durable contrast signals, contributing to their penetration beyond blood vessels and long-time retention in liver. Interestingly, we found that PEG-GVs did not exhibit a continuously enhanced accumulation in the liver tumor due to the EPR effect, but displayed a rapid regression. The long-time retention of PEG-GVs in normal liver tissue and rapid regression from liver tumor lead to distinct display of liver tumor boundaries, enabling the early diagnosis of small liver metastases and presenting advantages in guiding radiofrequency ablation of liver tumor. Moreover, we have also verified that PEG-GVs exhibit excellent imaging performance and biosafety in macaques. Our study provides new insights into the roles of PEG-GVs in liver tumor diagnosis and ablation guidance.

**Supplementary Information:**

The online version contains supplementary material available at 10.1186/s12951-025-03377-z.

## Introduction

Ultrasound has served as a visual tool for medical diagnosis and therapy due to its great advantages such as non-radiation, low cost, high spatiotemporal resolution, and deep tissue penetration [1, 2]. Despite the improvement of ultrasound resolution and image quality, there are still limitations in detecting small lesions and low-speed blood flow signals, which occasionally leads to misdiagnose. The application of ultrasound contrast agents (UCAs) provides an innovative approach to enhance the sensitivity and specificity of the ultrasonic examination. Technically, UCAs increase the difference of acoustic impedance between tissues or within vascular / tissue interfaces, augmenting blood perfusion of the lesion, which facilitates early-stage and precise diagnosis [3]. Meanwhile, UCAs also play an important role in guiding interventional procedures, such as needle biopsy and ablative therapy [4]. Nevertheless, clinical interventional therapy, such as radiofrequency ablation (RFA), often takes a considerable amount of time and requires repeated administration of UCAs. This brings to inconvenient clinical operations and increases the medical burden.

Among various UCAs, Sonovue and Sonazoid are the two most prevalently used in clinical applications [5, 6]. Both of them have gas core stabilized by lipid shells and can provide strong echo signals. Nevertheless, they present dissimilarities. For instance, in hepatic contrast-enhanced ultrasound (CEUS), Sonovue can be classified into three phases based on their perfusion characteristics: the arterial phase, the portal venous phase, and the late phase. Approximately five minutes after injection, Sonovue microbubbles are gradually eliminated from the hepatic parenchyma. Because of the rapid imaging regression of Sonovue, repeated injections are often needed in clinical settings [7]. Sonazoid can be specifically absorbed by Kupffer cells in the hepatic sinuses, leading to a post-vascular stage imaging, namely the Kupffer phase [8]. The Kupffer phase commences 5–10 min and lasts nearly 2 h [9, 10]. Previous studies revealed that the quantity of Kupffer cells in liver cancer tissue is conspicuously lower than that in non-cancerous liver tissue, offering valuable information in the differential diagnosis and the histological grading [11–13]. But some studies have also suggested that part of focal liver lesions, like atypical hemangioma, are easily misdiagnosed as HCC due to lack of Kupffer phase [14].

It is worthy of note that the aforementioned clinical UCAs were microbubbles and confined in circulation due to their microscale particle size, disabling them to pass through the tumor vessel for extravascular tissue imaging. In recent years, nanobubbles have been elaborately developed and thoroughly explored for extravascular tissue or cell imaging [15, 16]. Owing to their nanoscale particle size, they exhibit stronger tissue penetration and longer blood circulation time. To date, most nanobubbles are synthesized by chemical synthesis routes, which brings with some obstacles to clinic translation [17]. For instance, the fabrication procedure is intricate and difficult to produce on a large scale. Some UCAs are composed of inorganic silica and gold nanomaterials, leading to the concerns about potential toxicity and in vivo degradation [18, 19]. In recent years, gas vesicles (GVs), a kind of natural nanostructures derived from *hydrohaline archaea* or *cyanobacteria*, have been reported as ultrasonic contrast agents, with gas-filled protein shells and nanoscale size in the range of 44–600 nm [20, 21]. Our previous studies have demonstrated that GVs exhibit excellent acoustic echo signals and strong tissue-penetrating capability [22]. Nevertheless, no studies have been conducted to comprehensively compare GVs with the commercially available UCAs, especially their imaging performance in liver tumor.

**Fig. 1:**
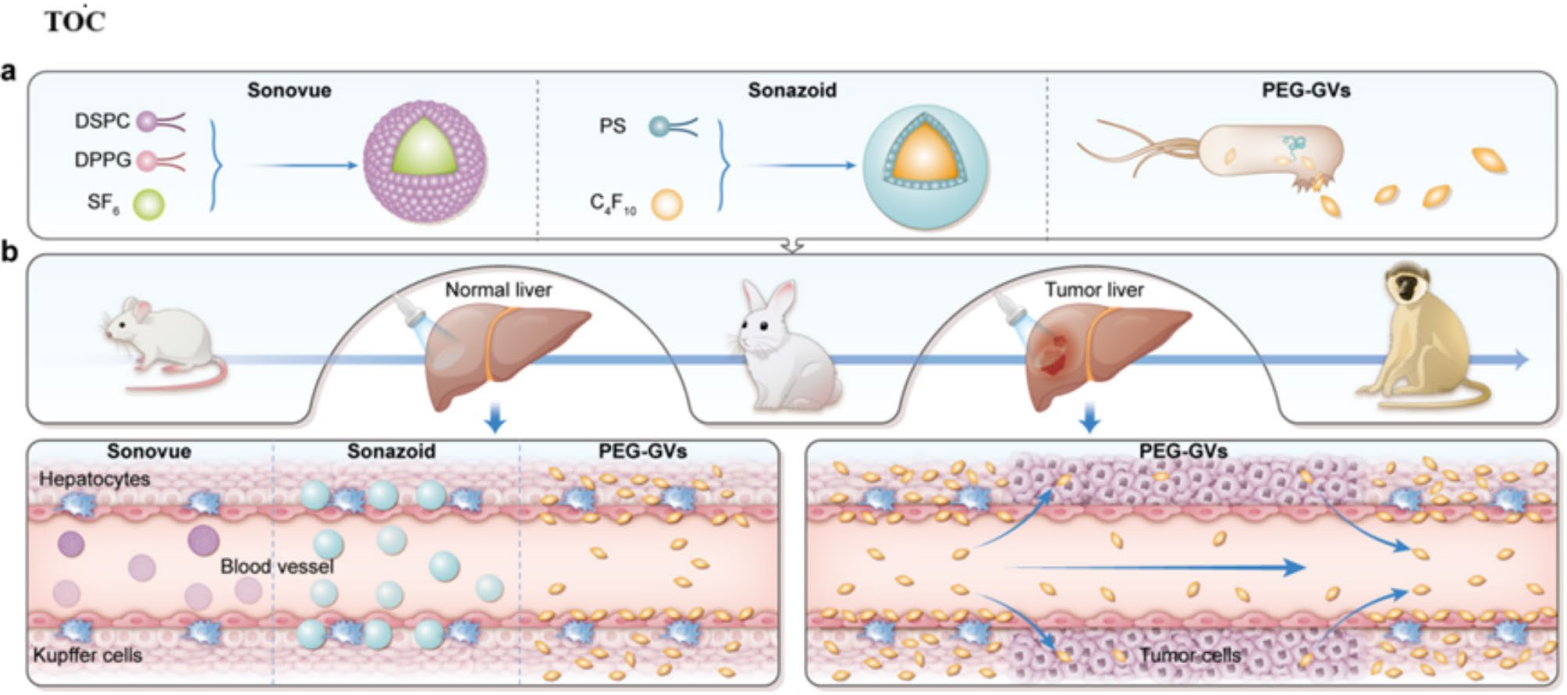
Schematic of commercial microbubbles and biosynthetic PEG-GVs for liver and liver tumor penetration. **a**, Schematic diagram of commercially available microbubbles and biosynthetic GVs. **b**, Mechanism diagrams of the vascular permeability of commercial microbubbles (Sonovue and Sonazoid) and biosynthetic nanobubbles (PEG-GVs). Left panel shows PEG-GVs but not Sonovue and Sonazoid can adhere and penetrate beyond blood vessels, providing long-duration signal enhancement in normal liver. Right panel demonstrates that PEG-GVs have a rapid regression from the tumor but long-time retention in normal liver, making it possible produce the distinct tumor boundary for liver tumor diagnosis and imaging-guided treatment.

In this study, we comprehensively compared PEGylated GVs (PEG-GVs) with Sonovue and Sonazoid in murine, rabbit and monkey models, involving in their physical and chemical properties, acoustic echo reflectivity, in vitro and in vivo imaging performance in liver and liver tumors, and the guiding role in radiofrequency ablation of liver cancer. Especially, we also evaluated the biosafety of PEG-GVs in mice and macaques. Significant differences in dynamic characteristics have been demonstrated, revealing that PEG-GVs possess superior stability and penetrability in liver imaging compared to commercial contrast agents Sonovue and Sonazoid. Moreover, PEG-GVs exhibits the property of adhering to the blood vessel walls in the liver. As a result, PEG-GVs offer a longer imaging duration. Additionally, we also found that PEG-GVs do not adhere to tumor blood vessels and displayed rapid regression in liver tumors. The long-time retention of PEG-GVs in normal liver tissue and their rapid regression from liver tumors lead to excellent postvascular phase imaging performance, which greatly facilitates the display of liver tumor boundaries and guides ablation treatment in real time (Fig. 1).

## Materials and methods

### Cell culture

LS-174T and VX2 cells were cultured in RPMI-1640 media containing penicillin (100 U/ml), streptomycin (100 µg/ml), and heat-inactivated fetal bovine serum (FBS, 10%).

## Construction of animal models

### Experimental animal source

The macaque, rabbits, and C57BL/6J and BALB/c nude mice used in the experiments, were purchased from Hubei Tianqin Biotechnology Co., Ltd., ZHUHAI BESTEST BIO-TECH CO., LTD and. Shenzhen LingFu TopBiotech., LTD., respectively.

### Murine liver metastasis model

The LS-174T Cells (about 1 × 10^5^) were surgically injected into the capsule of spleen through an abdominal incision under anesthesia in BALB/c nude mice. After injection, the spleen was gently returned to abdominal cavity and homoeostasis was resumed. The tumors’ growth were continuously monitored using a high-resolution ultrasound instrument for small animals (Visual Sonics VEVO 2100) until tumors grow to a size of 2–3 mm. About half month later, metastatic lesions can be observed by ultrasound.

### Rabbit orthotopic liver cancer model

Firstly, a xenograft tumor model was established by subcutaneous injection of 5 × 10^6^ VX2 cells into the right hindlimb of rabbits. When the tumor grows to 2–3 cm, the solid tumor block was excised and dissected under aseptic conditions, washed with normal saline (NS), and then excised into blocks of approximately 2-3 mm³ in size using ophthalmic scissors. Subsequently, these blocks were placed in a sterile dish. After the rabbits had been anesthetized, a 16 G puncture needle was inserted into the liver parenchyma under ultrasound guidance. Then the needle core was pulled out, and the tumor fragments were put into the needle sheath. The tumor fragments were pushed into the liver parenchyma by the needle core. Subsequently, the absorbent gelatin was immediately injected to seal the puncture site, then the needle was pulled out and the area was manually gently pressed for about 3 min. About 14 days later, when the tumor was detected by ultrasound, with a diameter of 3–5 mm, CEUS was performed.

### Preparation, functionalization, and characterization analysis of UCAs

The GVs were synthesized as previously reported [23]. PEG-GVs were prepared by covalently coupling polyethylene glycol (PEG5000) to the shell of GVs. Briefly, an appropriate amount of EDC (1 mg) and NHS (1.5 mg) were dissolved in 1 ml PBS solution (pH = 7.4). After fully dissolving, 1 ml of GVs (OD500 3.0) was added to the solution and placed on a shaker to gently oscillate for 2 h. Subsequently, the activated solution was slowly added to the pre-prepared PEG5000 solution and stirred overnight at 4 °C. Then, the GVs solution was centrifuged and washed with PBS four times to obtain the PEG-GVs. The particle size and zeta potential of GVs, PEG-GVs and commercial UCAs (Sonovue and Sonazoid) were measured using a particle size analyzer (ZS XPLORER). The morphology of PEG-GVs and commercial UCAs were also observed using a transmission electron microscope (TEM, GEM-F200) or an inverted fluorescence microscope (Leica).

### Fluorescence-labeled contrast agents

Sonovue and Sonazoid were prepared according to the instruction provided by the manufacturer. Dio fluorescent dye (40725ES10) (Yeasen, China) was stained the microbubble suspension and followed by incubation for 15–20 min at dark. After centrifugation at 200 g for 10 min, these microbubbles were collected and washed for three times to remove the free dye. PEG-GVs were fluorescently labeled using Sulfo-Cy3 NHS ester through covalent conjugation. The labeled PEG-GVs were then purified through 250 g centrifugation and PBS washing for 3–4 times to remove unreacted dye. Finally, the fluorescence labeled contrast agents (CY3-PEG-GVs, DIO-Sonazoid, and DIO-Sonovue) were obtained and stored at 4 °C.

### In vitro imaging and stability test

2% Agar powder was used to prepare the in vitro imaging mold. The vesicle optical density (OD) measured by a spectrophotometer at the wavelength of 500 nm (Nanodrop 2000c Thermo scientific, USA) was used as the concentration of PEG-GVs. In brief, ten microliters of PEG-GVs at different concentrations (with OD500 values of 1.0, 1.5, 2.0, 2.5, 3.0) and 90 µL of 1% hyaluronic acid were thoroughly mixed and then added to the agar mold. The microbubbles of Sonovue and Sonazoid were prepared according to the operating instructions. The prepared solutions were diluted with 0.9% normal saline until they exhibited a comparable CEUS imaging intensity to that of the corresponding concentration of PEG-GVs. CEUS was performed in contrast mode using an ultrasound instrument (Resona 9, Mindray, China), superficial probe (L11-3U), with imaging parameters as follows: transducer transmit frequency = 7.1 MHz, frame rate = 10 frames/s, gain = 65 dB, dynamic range = 115, MI = 0.072 (Sonovue), 0.106 (Sonazoid), 0.145 (PEG-GVs), respectively.

### In vivo imaging

After the experimental animals were anesthetized, the animals were placed on the 37 °C thermal insulation pad and fixed with tape in the supine position. After the optimal observation section was obtained by ultrasound and the Sonovue, PEG-GVs and Sonazoid were injected intravenously into the animals sequentially at 30-min intervals. To facilitate the comparison of the in vivo imaging performance of three contrast agents, we adjusted the injected doses to ensure that their peak intensities were comparable. In mouse experiments, 50 µL (stock solution diluted 3-fold), 35 µL (stock solution diluted 30-fold), and 100 µL (OD 3.0) were used for Sonovue, Sonazoid and PEG-GVs, respectively. In rabbit experiments, 80 µL (stock solution), 800 µL (OD 3.0) were used for Sonazoid and PEG-GVs, respectively.

The clinical ultrasound system (Resona 9, Mindray, China) equipped with a linear array transducer (L11-3U for mice and rabbits) or convex array transducer (SC-1U for macaques) were used. In the in vivo mouse and rabbit studies, the ultrasound imaging parameters were set as follows: 7.1 MHz transmit frequency, 10 Hz frame rate, 65 dB gain, 115 dynamic range. The mechanical index and acoustic power were set at 0.072, 1.29% for Sonovue, 0.106, 2.95% for Sonazoid and 0.145, 5.13% for PEG-GVs, respectively. In the macaque studies, the ultrasound imaging parameters were set as follows: 7.1 MHz transmit frequency, 10 Hz frame rate, 65 dB gain, 100 dynamic range, 4.79% acoustic power. The mechanical index was set at 0.227 for PEG-GVs. In all experiments, the acquisition time was approximately 15 min.

### Radiofrequency ablation

After muscle anesthesia, the rabbits were placed on the imaging platform to expose the skin of the abdomen and lower chest. After the optimal image was obtained by ultrasound, the probe was fixed and the UCAs were injected intravenously with 0.08 ml Sonazoid solution (prepared at the clinical dose) or 0.8 ml PEG-GVs (OD500 = 3.0). After the tumor boundary was displayed by CEUS, the radiofrequency ablation needle was inserted into the tumor under the guidance of ultrasound. When ultrasound showed that the high echo generated by ablation completely covered and slightly beyond the boundary of the tumor, ablation was stopped. The next day, CEUS was performed again to observe the condition of the lesions. After Systemic administration of UCAs, the area of ablation with no enhancement was regarded as deactivated, and it is unnecessary to ablate again. The ablation parameters were performed at 20 W for 1 min with an interval of 25 s. Finally, the complete ablation was confirmed by histopathology.

### Biosafety evaluation in the macaques

We examined the biosafety of PEG-GVs in 6 macaques. In the first week, 0.5 ml/kg PEG-GVs (OD 3.0) or equal volume of normal saline were intravenously injected into these macaques (*n* = 3). The contrast imaging performance of liver were evaluated at day 0 and the blood samples were collected at the day − 1, day 3 and day 7 for biosafety assay. The liver/kidney function and blood count were assessed. After one month, 1.0 ml/kg or 1.5 ml/kg PEG-GVs were intravenously injected into these macaques (*n* = 3). The contrast imaging performance of liver were evaluated at day 31 and the blood samples were collected at the day 30, day 34 and day 38 for biosafety assay. Still one month later, two macaques were injected 7.5 ml/kg PEG-GVs (5-fold high dose) or equal volume of normal saline (*n* = 1). The macaques were euthanized after 3 days for collecting the main organs including heart, liver, spleen, lung, and kidney tissues. Hematoxylin-eosin (H&E) staining analysis was performed according to the standard protocol. Briefly, these organs were fixed in 4% paraformaldehyde, processed through dehydration, clearing, and paraffin embedding. Five-micrometer sections were cut, deparaffinized, rehydrated, and stained with hematoxylin-eosin dyes. Histopathological changes were observed under light microscopy. All procedures were conducted in an aseptic operating room with isoflurane anesthesia.

### Tissue Immunofluorescence analysis

Briefly, the fluorescence-labeled Sonovue, Sonazoid or PEG-GVs (100 µl, OD500 = 3.0) were intravenously injected into tumor-bearing mice, respectively. Then, the mice were sacrificed according to the required time points. After that, the tumors were removed for frozen sections, and then stained with anti-CD31 antibody (GB113151) (Servicebio, China) for vascular cells, the anti-F4/80 antibody (GB113373) (Servicebio, China) for intrahepatic Kupffer cells, and observed with a confocal laser scanning microscope (A1R, Nikon, Japan).

### Statistical analysis

In this study, GraphPad Prim 9.0 was used for data analysis. The normality of the data is judged by Shapiro-Wilk test, and the data in accordance with the normal distribution is presented in the form of mean ± standard deviation, otherwise as standard deviation. The differences between groups were detected by bilateral Student t test or Mann-Whitney test. Multiple groups of data were compared by single factor analysis of variance. *P* < 0.05 was considered to be statistically significant.

## Results and discussion

### Preparation and characterization of PEG-GVs

**Fig. 2:**
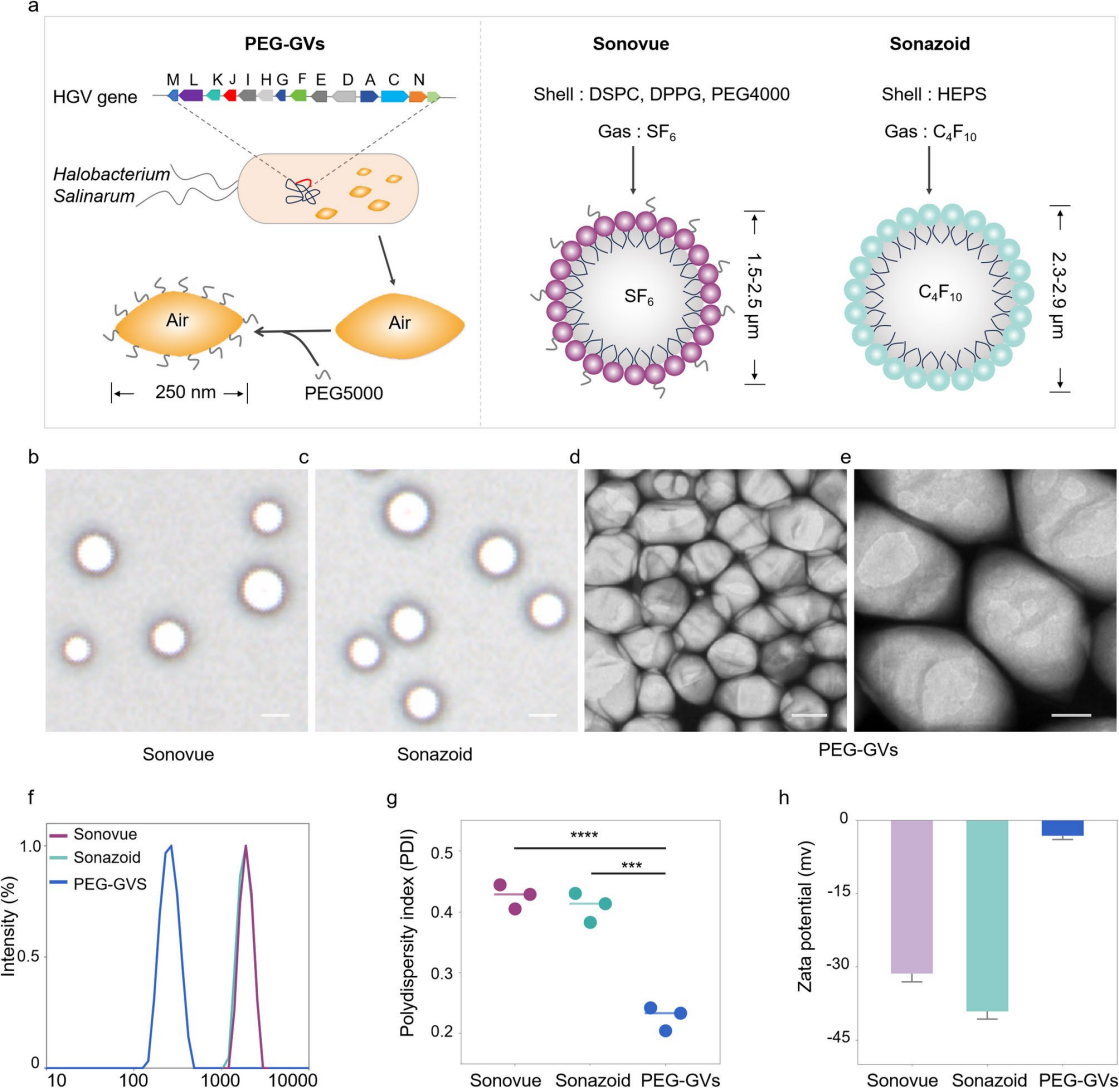
Preparation and characterization of PEG-GVs. (**a**) Composition and structure diagram of biosynthetic PEG-GVs, Sonovue and Sonazoid. **b**-**c**, the morphologies of Sonovue (**b**) and Sonazoid (**c**) observed under microscope. Scale bar: 2.5 μm. **d**-**e**, SEM images of PEG-GVs. Scale bar: 100 nm (**d**), 50 nm (**e**). **f**-**h**, Particle size distribution (**f**), PDI (**g**) and Zeta potential (**h**) of PEG-GVs, Sonovue and Sonazoid. *** *p* < 0.001, **** *p* < 0.0001

Unlike Sonovue and Sonazoid produced through chemical synthesis with lipid components and inert gases like sulfurhexafluoride (SF_6_) or perfluorobutane (C_4_F_10_), GVs are synthesized via a biological synthesis route. Generally, GVs are encoded by gvp gene clusters in the genome and assembled into gas-filled protein shells along with the growth of microorganisms. Firstly, we extracted GVs from *Halobacterium Salinarum* through lysis method as reported previously [24–26]. To reduce their immunogenicity to body, PEG5000 was subsequently conjugated onto the surface of GVs through amidation reaction in the presence of EDC and NHS, resulting in the formation of PEG-GVs (Fig. 2a, left panel). The structure and composition of PEG-GVs also diverge significantly from Sonovue and Sonazoid. PEG-GVs feature gvp protein shells and air core. In contrast, Sonovue is composed of DSPC/DPPG shells encapsulating an SF_6_ core, and Sonazoid predominantly consists of hydrogenated egg phosphatidyl serine (HEPS) shells with a C_4_F_10_ core (Fig. 2a, right panel) [27]. This structural disparity is also reflected in their morphological appearance. Under the microscope, Sonovue and Sonazoid both exhibit a spherical shape, while PEG-GVs display an ovary shape under the scanning electron microscope (SEM) (Fig. 2b-2e). The hydrodynamic size of the UCAs was also measured via dynamic light scattering (DLS). Notably, GVs and PEG-GVs are significantly smaller, with a mean hydrodynamic diameter of 213.60 ± 1.71 nm, 252.90 ± 3.08 nm, respectively (Figure S1), as opposed to Sonovue and Sonazoid which have particle sizes in the micrometer range: 2.18 ± 0.78 μm and 2.36 ± 0.95 μm, respectively (Fig. 2f). The polydispersity index (PDI) further differentiates these agents. The value of this index for PEG-GVs is 0.23 ± 0.02, which is substantially lower than that of Sonovue 0.43 ± 0.02  and Sonazoid 0.41 ± 0.02 (Fig. 2g). In our in vitro observation experiments, we also found that compared to Sonovue and Sonazoid, PEG-GVs exhibit superior dispersion characteristics and long-term stability (Figure S2). The zeta potentials of PEG-GVs, Sonovue, and Sonazoid are measured at -3.17 ± 0.78 mV, -31.38 ± 1.69 mV, and − 39.16 ± 1.55 mV, respectively (Fig. 2h). Thus, our results demonstrate that PEG-GVs have uniform nanoscale particle size and outstanding stability.

### Acoustic characteristics and in vitro imaging of PEG-GVs

**Fig. 3:**
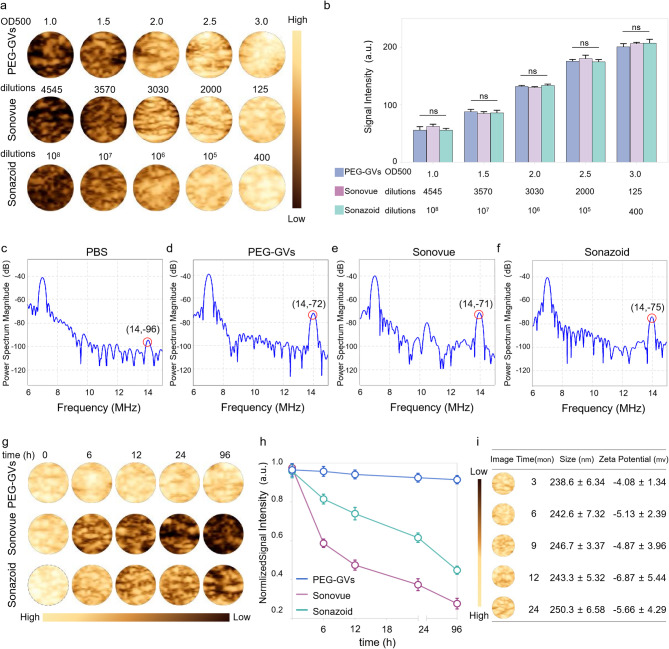
Acoustic characteristics and in vitro imaging of PEG-GVs. a-b, In vitro nonlinear contrast imaging (a) and quantification (b) of PEG-GVs, Sonovue and Sonazoid at the different concentrations. c-f, In vitro nonlinear signal spectra of PBS solution (c), PEG-GVs (d), Sonovue (e) and Sonazoid (f). g-h, The contrast images (g) and quantification (h) of the three UCAs kept at 4°C for different time from 0 h to 96 h. i, In vitro nonlinear contrast imaging, particle size, and zeta potential of PEG-GVs kept at 4°C for 3, 6, 9, 12, 24 months.

To facilitate comparison of PEG-GVs with Sonovue and Sonazoid in subsequent experiments, we first optimized the concentrations of PEG-GVs to achieve a comparable imaging intensity to Sonovue and Sonazoid. As depicted in Fig. 3a-3b, we can see that the CEUS imaging intensities of PEG-GVs at OD500 1.0, 1.5, 2.0, 2.5 and 3.0 were comparable with Sonovue at 4545-, 3570-, 3030-, 2000-, 125-fold dilutions and Sonazoid at 10^8^-, 10^7^-, 10^6^-, 10^5^-, 400-fold dilutions, respectively. Generally, nonlinear imaging modes depend on harmonic signals from microbubble or nanobubble oscillations excited by ultrasound waves, improving contrast specificity of CEUS. Especially, the second harmonic signals are crucial for CEUS imaging. Therefore, we compared the harmonic signal characteristics of PEG-GVs, Sonovue and Sonazoid. Transmitting at 7 MHz, we observed substantial second-harmonic signals in PEG-GVs at 14 MHz compared to PBS control. As expected, Sonovue and Sonazoid produced substantial second-harmonic signals at 14 MHz different from Sonazoid and PEG-GVs, Sonovue also produced ultraharmonic signals (3/2 f0) at 10.5 MHz (Fig. 3e). This phenomenon mainly arises from flexible and nonlinear deformable phospholipid shells and was also observed by *other* group [28]. Quantitative analysis showed the second harmonic signals of Sonovue, Sonazoid and PEG-GVs had 25.85 ± 1.70 dB, 22.57 ± 3.03 dB, and 24.00 ± 1.29 dB higher power spectrum magnitude than the PBS solution at the transmitted frequency (Figure S3). Notably, PEG-GVs produced comparable nonlinear second-harmonic signal intensities, similar with the commercial Sonovue, Sonazoid. To compare the imaging stability of these UCAs, PEG-GVs (OD500 3.0) and Sonovue and Sonazoid (at corresponding concentrations with comparable CEUS imaging intensities) were kept at 4 °C and imaged at different time intervals (Fig. 3g-h). The results showed that the contrast imaging intensity of Sonovue rapidly declined to below 50% in several hours and below 30% after 96 h, while that of Sonazoid declined more slowly but also dropped below 50% at 96 h. In stark contrast, PEG-GVs showed no significant signal decrease during the 96-h observation period (Fig. 3h). Additionally, it was also observed that Sonovue and Sonazoid significantly vanished over time, while the PEG-GVs largely retained their original signal intensity (Fig. 3g), indicating the excellent imaging stability of PEG-GVs relative to commercial Sonovue and Sonazoid. To further assess the long-term stability of PEG-GVs, we extended the observation period to 24 months and detected GVs’ imaging performance, particle size and zeta potential. We found that PEG-GVs still remained stable in imaging performance, particle size and zeta potential throughout 24-month observation period (Fig. 3i). These results indicate that PEG-GVs kept excellent CEUS imaging performance and robust stability, facilitating their biomedical application and long-term preservation.

### *In vivo* CEUS imaging of PEG-GVs in normal liver tissue 

**Fig. 4:**
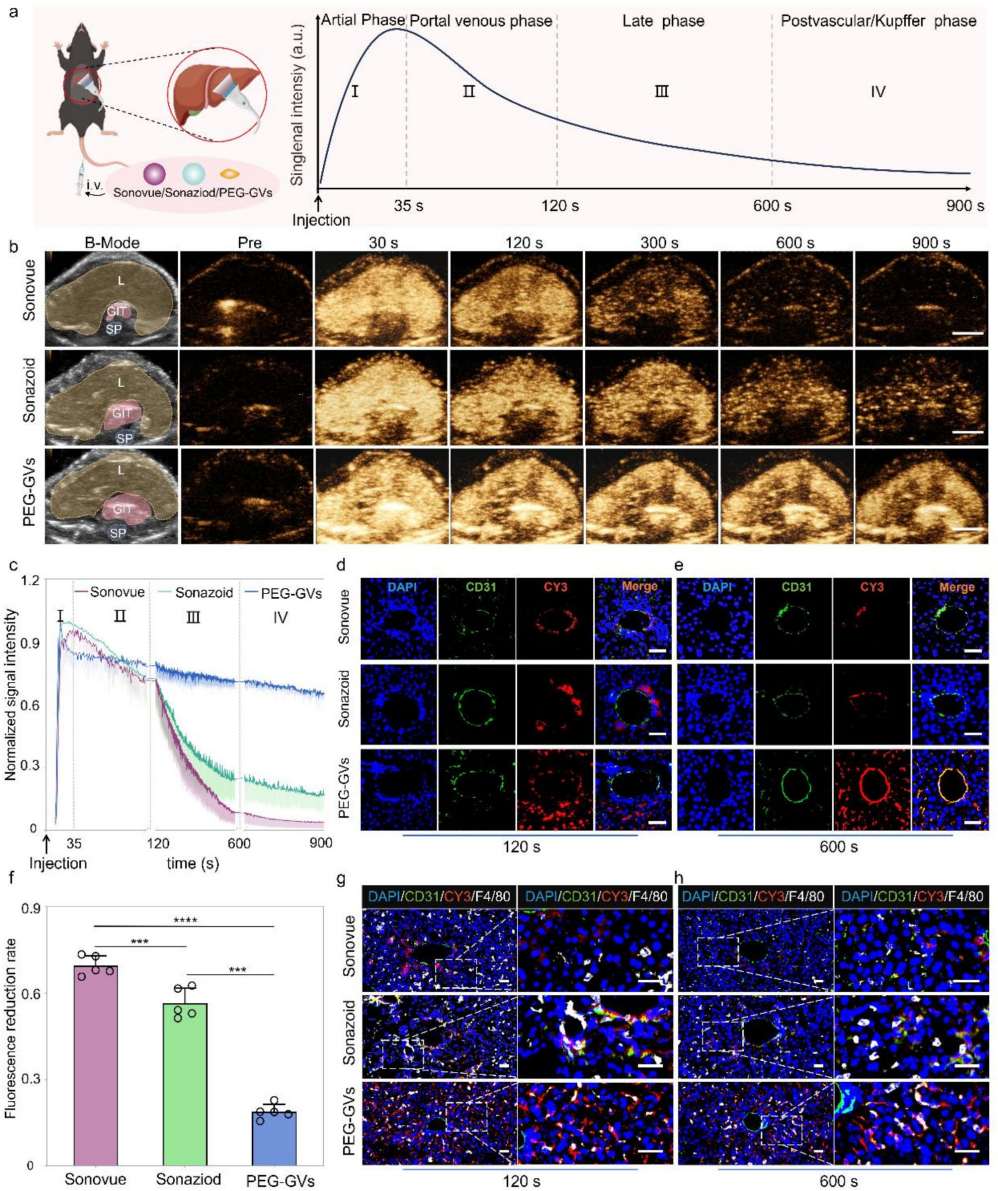
CEUS imaging performance and mechanisms of PEG-GVs in normal liver. **a**, Diagram of normal liver CEUS and its four phases when imaging of liver in vivo, scale bar: 5 mm. **b**, The CEUS images of different phases in the same mice after systemic administration of Sonovue, Sonazoid and PEG-GVs at 30 min intervals. The “L”, “GIT”, “SP” represents liver, stomach and intestines, and spine on the B-mode images, respectively. **c**, The normalized signal intensity curves of CEUS images in Fig. 4b. **d**-**f**, Immunofluorescence images of vessels (stained with anti-CD31) and CY3-labeled UCAs at 120 s (**d**) and 600 s (**e**) after systemic administration. f, The quantification of fluorescence reduction rate in Fig. 4d. Scale bar: 40 μm. *** *p* < 0.001, **** *p* < 0.0001.(*n* = 5), **g**-**h**, Immunofluorescence images of liver tissues at 120 s (**g**) and 600 s (**h**) after systemic administration. Kupffer cells were stained with anti-F4/80 and UCAs were stained by CY3 dye. Scale bar: 40 μm.

Based on the outstanding stability and imaging performance of PEG-GVs in vitro, we further compared the liver contrast imaging performance of PEG-GVs with Sonovue and Sonazoid in healthy C57BL/6J mice. The experimental process and phases of ultrasound contrast agent infusion are depicted in Fig. 4a, showing the typical contrast echo signal enhancement modes in the arterial phase, portal venous phase, late phase and postvascular phase (also called Kupffer phase for Sonazoid). To reduce individual differences, we intravenously administered these UCAs in the same mice at 30-min intervals, followed by a short high-power burst before next injection. As shown in Fig. 4b, the echo signals of liver received with Sonovue were enhanced in the arterial phase (30 s), subsided in the portal venous phase (120 s), and weakened rapidly after 300 s. Sonazoid has a prolonged imaging time in Kupffer phase and provides more details for liver disease diagnosis. Our results show that the liver echo signal of Sonazoid was significantly enhanced in the arterial phase (30 s), slightly reduced in the delayed phase (300 s), significantly decreased in the Kupffer phase (600 s), and becoming weak after 900 s. Interestingly, the liver echo with PEG-GVs remained stable across all phases, much stronger than Sonovue and Sonazoid, especially in the late and Kupffer phases (Fig. 4b). Quantitative data further revealed that the imaging intensity of Sonovue and Sonazoid declined rapidly subsequent to the portal phase (120 s), whereas PEG-GVs remained at a high level, with a decline rate of less than 20% in 600 s (Fig. 4f) and still less than 30% until 900 s (Fig. 4c). The normalized AUC (Area under curve) 900 s of PEG-GVs was greater than that of Sonazoid and Sonovue, being 2.72- times of Sonazoid and 1.82 times of Sonovue, respectively (Figure S4). Obviously, this prolonged imaging duration offers longer time for analysis, thereby leading to more precise diagnosis and efficient interventional operation for doctors.

To further elucidate the mechanisms underlying prolonged imaging duration, we performed immunofluorescence staining using CY3-labeled PEG-GVs and DIO-labeled Sonazoid and Sonovue. Immunofluorescence staining of liver sections revealed a considerable amount of PEG-GVs penetrated beyond blood vessels and achieved liver cells. In contrast, scarce Sonazoid and Sonovue could infiltrate blood vessels and settled around blood vessels. Notably, we witnessed the progressive adhesion of PEG-GVs to the vascular walls after systemic administration. Bubble adhesion was still observable at 120 s and annular adhesion to the entire blood vessel wall at 600 s after injection (Fig. 4d, 4e, Figure S5). This PEG-GVs’ adherence to the blood vessel walls could be observed for up to 1 h (Figure S6), which might partly explain their prolonged imaging duration of liver. Considering Sonazoid’s distinct advantage in the Kupffer phase as UCAs, we further investigated Kupffer cell uptake of different UCAs in liver tissue. Our findings revealed that Sonazoid and PEG-GVs, but not Sonovue, were conspicuously internalized by Kupffer cells at 120 s and 600 s. Moreover, the uptake of PEG-GVs elevated over time (Fig. 4g, 4h). Collectively, our data indicated PEG-GVs possesses outstanding CEUS capability in the liver, which may attribute to their adhesion and penetration beyond blood vessels and phagocytosis by Kupffer cells.

### In vivo CEUS imaging of PEG-GVs in small liver tumor 

**Fig. 5:**
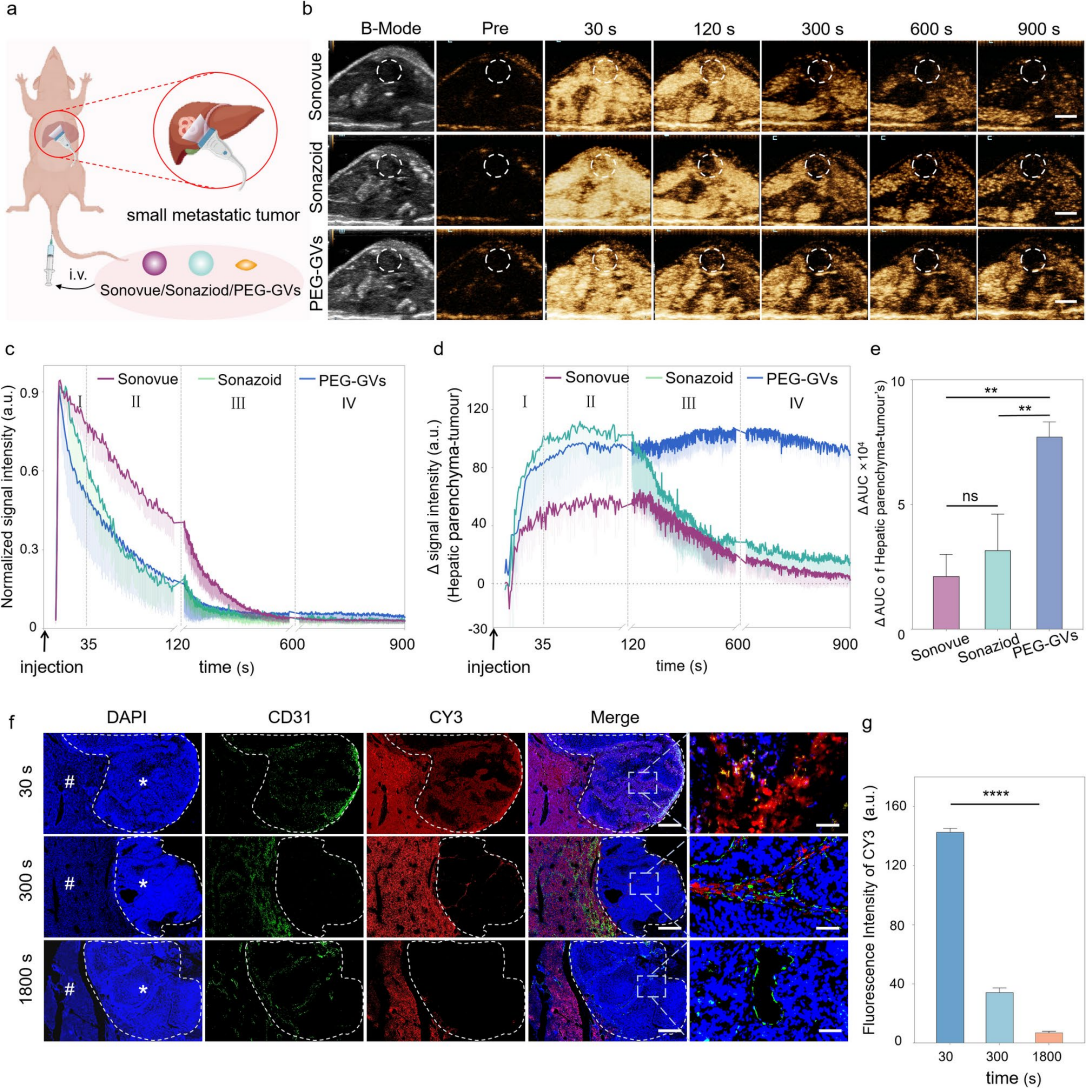
CEUS imaging performance and mechanism of PEG-GVs in liver tumor. a, Diagram of CEUS of three UCAs in small liver metastatic tumors. b, CEUS images of Sonovue, Sonazoid and PEG-GVs at different time points after systemic administration，scale bar: 5mm. c, The normalized signal intensity curves of tumors in Figure5b. d-e, CEUS signal intensity of peripheral liver tissue (d) and the quantification of AUC difference between the surrounding liver tissue and tumor at 900 s (e). f-g, Blood vessels immunofluorescence staining by anti-CD 31 (f) and quantification (g) of liver metastatic tumors at 30 s, 300 s, 1800 s after CY3-labeled PEG-GVs administration. # and * represent normal liver and tumor area, respectively. Scale bar: 1000 µm. **** p < 0.0001.

The Kupffer phase in liver CEUS has been demonstrated to enhance the detection rate and specificity of small liver tumor lesions [3, 29]. In light of the finding that Kupffer cells can phagocytose PEG-GVs, we hypothesized that they might play a significant role in the diagnosis of liver tumors. To test it, we employed a murine model of liver metastatic tumors to explore the possible value of PEG-GVs in detecting minuscule hepatic lesions (Fig. 5a). After the systemic administration, all the three UCAs exhibited rapid perfusion and rapid regression in the tumor (Fig. 5b-5c). Additionally, we observed a sustained high enhancement of PEG-GVs in the normal liver tissue, attributing to their vascular adhesion and Kupffer cell uptake as described above. Interestingly, PEG-GVs did not exhibit a sustained enhancement in liver tumor tissue due to EPR effects but regressed rapidly. This leads to a persistently lower imaging intensity of tumors relative to peripheral liver tissue, especially in the late phase and Kupffer phase (Fig. 5d). Quantitative analysis of tumor-subtracted hepatic parenchyma AUC revealed PEG-GVs had 3.62-fold and 2.44-fold higher than Sonovue and Sonazoid, respectively (Fig. 5e). The immunofluorescence results further confirmed that PEG-GVs did not accumulate in the tumor area over time but declined rapidly instead, while it scarcely decreased in the surrounding normal liver tissue. This might be attributed to the fact that PEG-GVs do not adhere to the vascular endothelium of tumor tissue, thereby failing to remain in the blood vessels for an extended period (Fig. 5f, Figure S7). Quantitative data also demonstrated that PEG-GVs in the tumor decreased to only 24.3% of peak intensity after 300 s of injection and less than 10% after 1800 s (Fig. 5g).

PEG-GVs are nanoscale with approximately 250 nm particle size while the endothelial space in neovascularized tumors is 380–780 nm, making them easily pass through the tumor vessel via the EPR effect [30, 31]. The imaging performance of PEG-GVs may help visualize the EPR process of these nanobubbles by ultrasound in a real time manner. Meanwhile, the rapid regression of PEG-GVs from liver tumor also challenges the traditional concept that the EPR effect causing from tumor leaky vessel does not necessarily prolong the retention time of nanoparticles in a tumor, at least for nanobubbles such as PEG-GVs. This unique phenomenon also renders it feasible for PEG-GVs to accurately define tumor boundaries, thereby facilitating early diagnosis. As for the mechanisms, the possible reasons might be attributed to the high permeability of tumor blood vessels, which endows these nanobubbles with higher penetrating capability into the tumor, but meanwhile it may also facilitate their easier washout from the tumor compared to the normal liver tissue. The structural defect of glycocalyx on the surface of tumor blood vessels may also contribute to the fast rapid regression of PEG-GVs from liver tumor [32, 33]. These results imply that PEG-GVs, based on their long-time retention in the normal liver and rapid regression in the tumor, can serve as an outstanding contrast agent in the diagnosis of early liver tumors and help to produce a distinct display of tumor boundaries. Moreover, we found that the enhanced contrast signal difference between the tumor and surrounding liver tissue can last up to 15 min (900s), which could be beneficial for imaging guided procedures such as minimally invasive treatments of liver tumors.

### Role of PEG-GVs in guiding liver tumor RFA in vivo

**Fig. 6:**
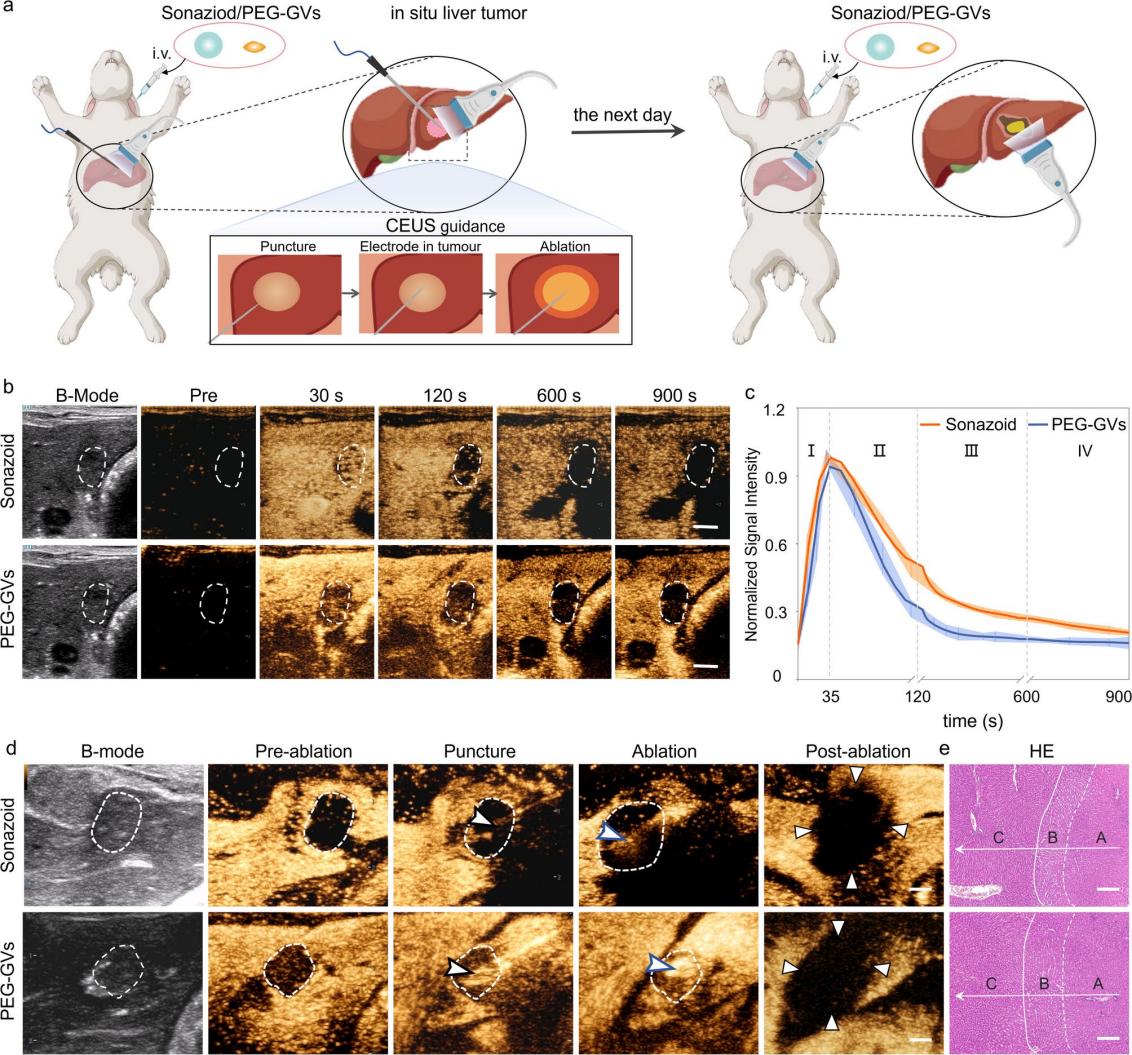
Role of PEG-GVs in guiding RFA of liver tumor. **a**, Diagram of and CEUS-guided RFA treatment after administration of Sonazoid or PEG-GVs in rabbits in situ liver tumor. **b**-**c**, CEUS images in different phases of rabbits in situ liver tumor (**b**) and normalized signal intensity curves (**c**), (*n* = 5). Scale bar: 5 mm. **d**, B-mode ultrasound images of preoperative tumor, revealing the unclear tumor boundary (white dotted circles). The tumor boundary could be seen in CEUS image till the portal venous phase (white dotted circles). With the CEUS guidance, monitoring puncture, radiofrequency electrodes (black arrowheads) were easily inserted into the index tumor. The electrode needle was turned on and ablation were performed (blue arrowheads). The CEUS was repeated after RFA ablation. Triangle points to the area of non-enhancement in the early arterial phase, indicating a necrotic tumor immediately after RFA. (*n* = 3). Scale bar: 5 mm. e, HE staining of the ablated tissue. A, B, C stand for coagulated necrotic liver tumor after RFA, peritumoral edematous liver tissue and normal liver tissue, respectively (*n* = 3). Scale bar: 200 μm.

Given the great potential of PEG-GVs in ultrasound imaging of the liver and liver tumors, we further investigated their role in guiding radiofrequency ablation (RFA) in an in situ rabbit liver tumor model (Figure S8a). As is well known, the distinct display of tumor boundaries is essential for determining tumor ablation extent. However, the tumor boundaries usually cannot be clearly identified on grayscale ultrasound. In contrast, CEUS can precisely locate the lesion, guide the puncture, and promptly evaluate the therapeutic effect during ablation. Considering that Sonazoid has been widely used in guiding RFA ablation, we conducted a comparison between Sonazoid and PEG-GVs for guiding ablation of liver tumor [34, 35]. Sonazoid or PEG-GVs were administered intravenously to rabbits with orthotopic VX2 tumors and then performed the ultrasound-guided RFA, followed by a second administration next day (Fig. 6a). As illustrated in Fig. 6b, both PEG-GVs and Sonazoid exhibited a broader spectrum of tumors compared to their two-dimensional B-mode images. Notably, the tumor range displayed by PEG-GVs was even larger and remained durable over time. The normalized signal intensity curves indicated that both PEG-GVs and Sonazoid exhibited rapid perfusion and regression in early liver tumors (Fig. 6c). However, PEG-GVs retained in the surrounding liver tissue for a longer period, producing more distinct tumor boundaries. The tumor boundaries displayed by PEG-GVs make it more appropriate for guiding precise tumor ablation, especially for guiding punctures and observing the ablation range by ultrasound in a real-time manner, as depicted in Fig. 6d. HE staining clearly revealed normal liver tissue, peritumoral edematous liver tissue, and coagulated necrotic liver tumor tissue after RFA, indicating that complete ablation was achieved in both groups (Fig. 6e and Figure S8b). These results suggest that PEG-GVs plays a similar role to Sonazoid in guiding RFA of liver tumors.

### CEUS imaging and biosafety of PEG-GVs in macaque

**Fig. 7:**
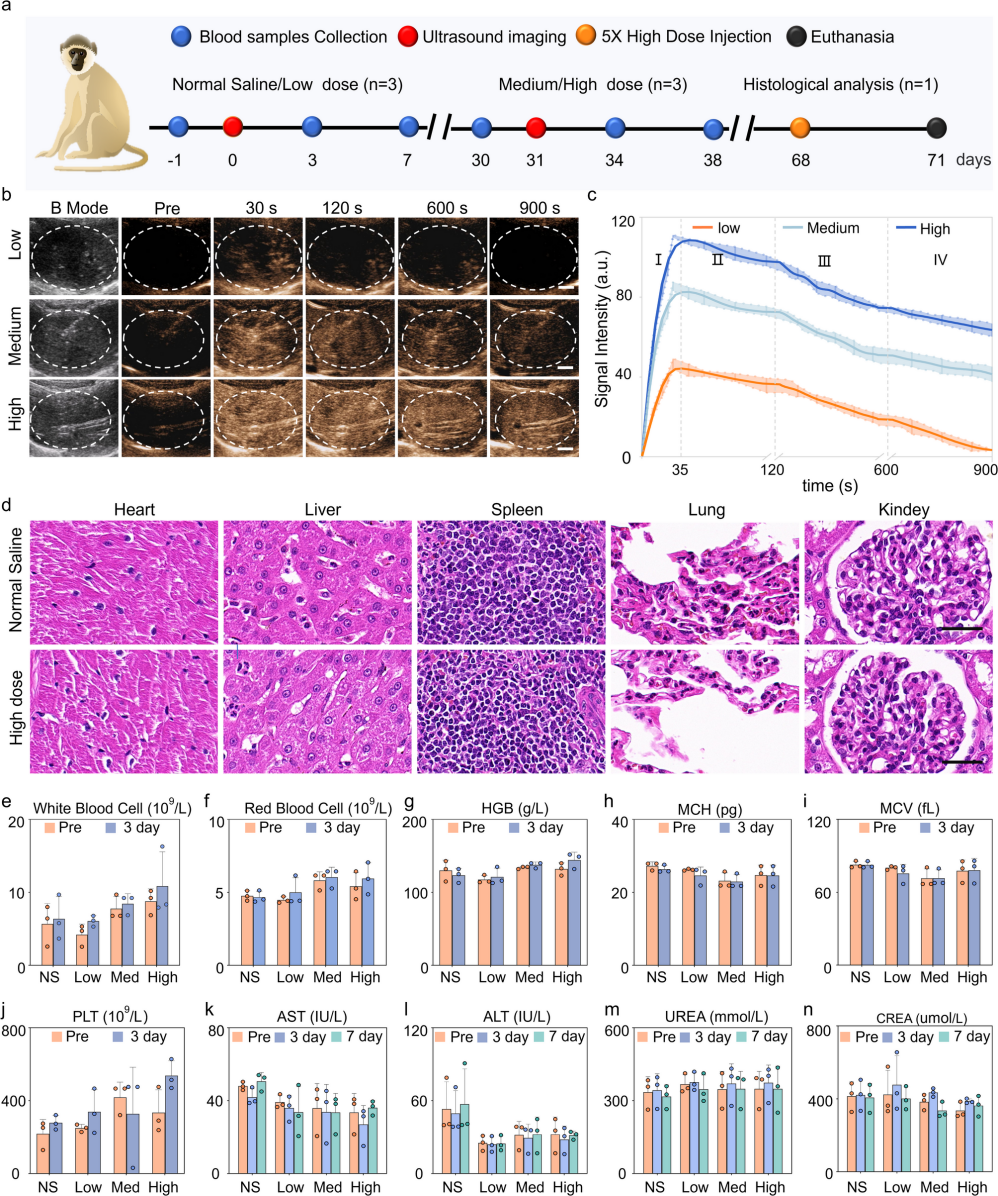
Liver CEUS imaging and biosafety of PEG-GVs in macaque. a, Schematic diagram of biosafety assessment in macaques. b-c, CEUS images of macaque liver (b) and the signal intensity curves of the three ultrasonic agents in four phases (c). d, HE staining of major organs of macaques received with normal saline or high-dose PEG-GVs. e-n. Hematological detection of each index before and after systemic administration of normal saline or PEG-GVs.

Macaques are highly similar to humans in terms of tissue structure, physiology, and metabolism. Hence, we further investigated the imaging performance of PEG-GVs in macaques. The experimental process is depicted in Fig. 7a. Figure 7b and c demonstrated that each dose of PEG-GVs may yield a rapid enhancement, reaching a peak about 35 s after systemic administration, then gradually decreased over time. With the increase of bubble doses, the contrast signal intensities increased and persisted for a longer duration. Notably, 15-min duration was exhibited for medium and high dose groups. Quantitative data indicated that the contrast signal intensities of three group at peak achieved 48.44 ± 3.70 a.u, 86.15 ± 3.11 a.u, 113.8 ± 3.66 a.u., respectively. It is worth noting that medium and high dose groups remained nearly half of the signal intensities after 900 s following the injection of PEG-GVs (Fig. 7c). To verify the in vivo biosafety of PEG-GVs, histological analysis of major organs and serum biochemical assays were conducted at the endpoint. HE staining data indicated that there was no significant damage to the heart, lung, kidney, and liver in the high dose macaque group (Fig. 7d), similar to the results obtained in mice (Figure S9). Tests of blood samples, including liver function, kidney function, and blood count, further indicated that PEG-GVs did not cause significant side effects (Fig. 7e-n, Figure S10). Therefore, PEG-GVs, as a novel ultrasound contrast agent, might hold broad prospects for future clinical applications.

## Conclusions

In summary, we developed a kind of novel biosynthetic nanobubbles (PEG-GVs) and comprehensively compared their physicochemical characteristics and contrast imaging performance with those of commercial Sonovue and Sonazoid microbubbles. Our results demonstrated PEG-GVs, thanks to their nanoscale size, may adhere to blood vessel walls and penetrate beyond blood vessels, producing more stable and durable contrast signals in liver. This property leads to significantly stronger contrast signals in the late phase and Kupffer phase than commercial Sonovue and Sonazoid. More importantly, we found that PEG-GVs did not exhibit continuous enhanced accumulation in the liver tumor due to the EPR effect, but displayed a rapid regression. The long-time retention of PEG-GVs in normal liver tissues and rapid regression from liver tumor greatly help the distinct display of liver tumor boundaries, enabling the early diagnosis of small liver metastases and presenting advantages in guiding radiofrequency ablation of liver tumor. A comparable effect in guiding RFA of liver tumors was achieved for PEG-GVs, similar to Sonazoid. Also, we confirmed PEG-GVs have excellent imaging performance and biosafety in macaque, paving the way for the future clinical translation of PEG-GVs.

## Electronic supplementary material

Below is the link to the electronic supplementary material.

Supplementary Material 1

## Data Availability

No datasets were generated or analysed during the current study.

## Abbreviations

UCAs: Ultrasound contrast agents
RFA: Radiofrequency ablation
CEUS: Contrast-enhanced ultrasound
GVs: Gas vesicles
PEG-GVs: PEGylated GVs
SF_6_: Sulfurhexafluoride
C_4_F_10_: Perfluorobutane
HEPS: Hydrogenated egg phosphatidyl serine
SEM: Scanning electron microscope
DLS: Dynamic light scattering
PDI: polydispersity index.
NS: Normal Saline
L: Liver
GIT: stomach and intestines
SP: Spine
