# Correction: PEGylated gas vesicles: a promising novel ultrasound contrast agent for diagnosis and guiding radiofrequency ablation of liver tumor

**DOI:** 10.1186/s12951-025-03491-y

**Published:** 2025-05-30

**Authors:** Kezhi Yu, Yongquan Huang, Yuanyuan Wang, Qunyan Wu, Zihang Wang, Fei Li, Jianri Chen, Maierhaba Yibulayin, Shushan Zhang, Zhongzhen Su, Fei Yan

**Affiliations:** 1 Department of Ultrasound, the Fifth Affiliated Hospital of Sun Yat-sen University, Zhuhai, Guangdong Province China; 2 Key Laboratory of Quantitative Synthetic Biology, Shenzhen Institute of Synthetic Biology, Shenzhen Institutes of Advanced Technology, Chinese Academy of Sciences, 1068 Xueyuan Avenue, Shenzhen University Town, Shenzhen, 518055 Guangdong Province China; 3 Department of Echocardiography, Xinjiang Key Laboratory of Ultrasound Medicine, First Affiliated Hospital of Xinjiang Medical University, Urumqi, Xinjiang Province China; 4 Research Center for Advanced Detection Materials and Medical lmaging Devices, Shenzhen Institutes of Advanced Technology, Chinese Academy of Sciences, Shenzhen, Guangdong Province China; 5 Southern University of Science and Technology, Shenzhen Guangdong Province, China

**Correction:** ***Journal of Nanobiotechnology*** **(2025) 23:344**

10.1186/s12951-025-03377-z

In this article Yongquan Huang, Zhongzhen Su, Fei Yan should have been denoted as a corresponding authors, instead of only Fei Yan.

The original article has been corrected.

