# Supplementary material for: PEGylated gas vesicles: a promising novel ultrasound contrast agent for diagnosis and guiding radiofrequency ablation of liver tumor
Source: J Nanobiotechnology. 2025 May 14;23:344. doi: 10.1186/s12951-025-03377-z (PMC12076873; doi:10.1186/s12951-025-03377-z)
Supplement: Supplementary file 1 — Supplementary Material 1 [file 12951_2025_3377_MOESM1_ESM.docx]

**Supplement Figures**


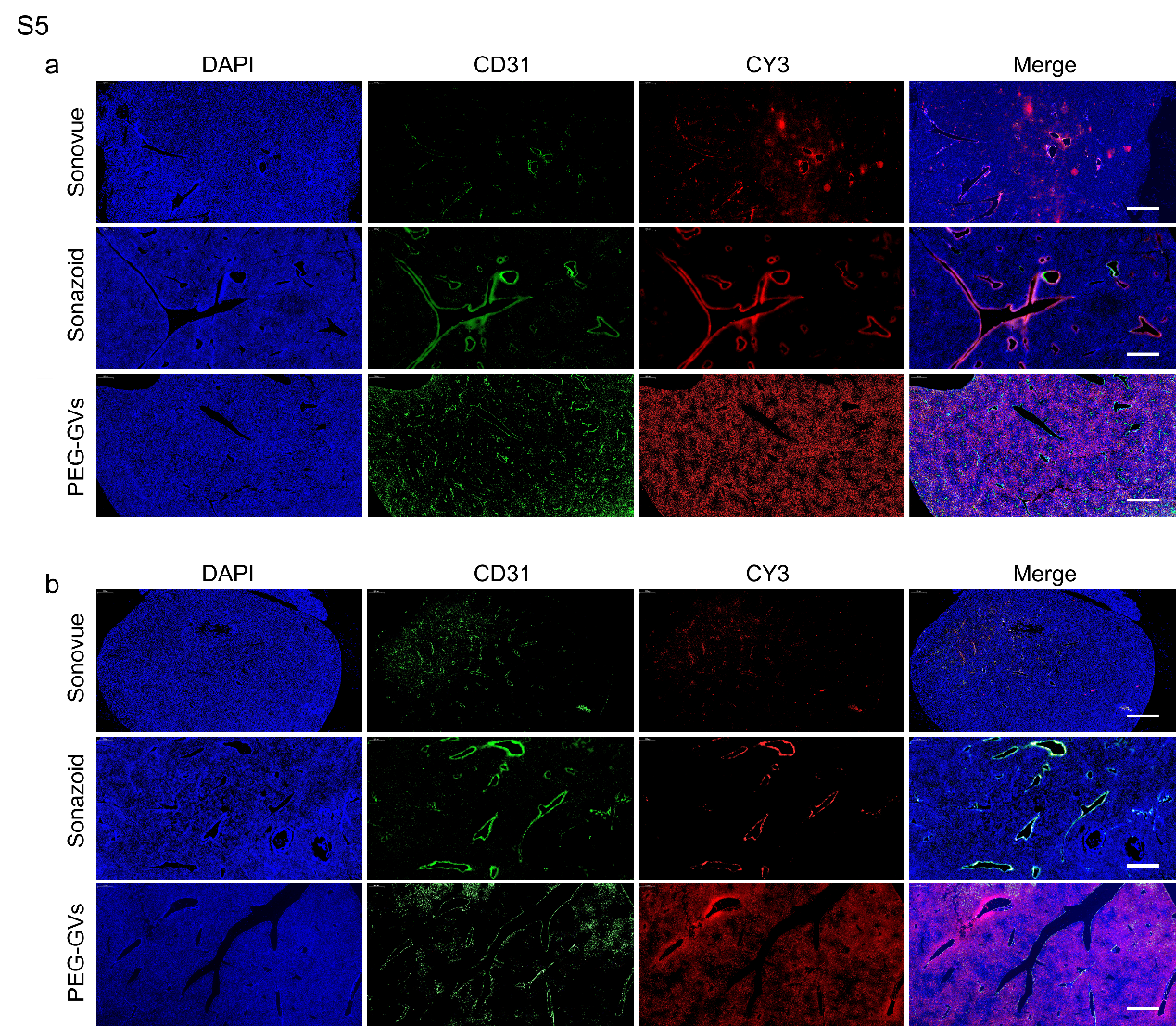

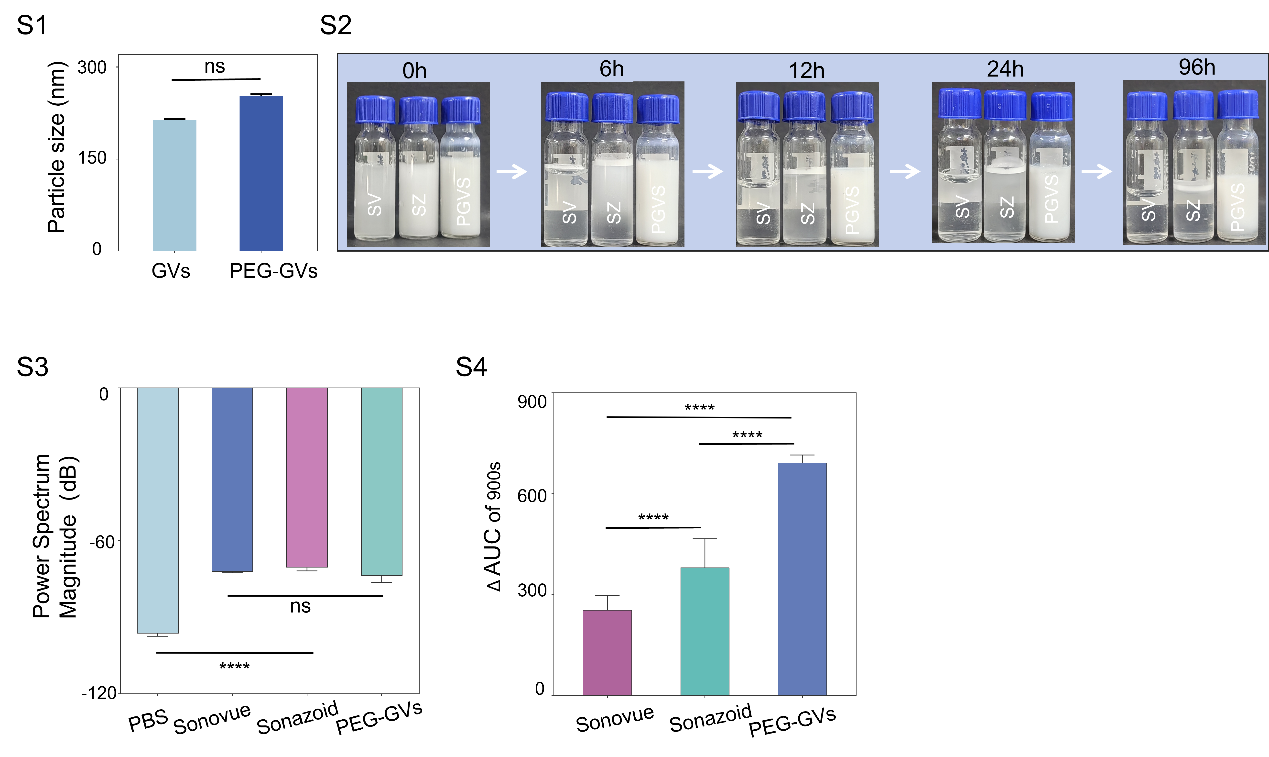


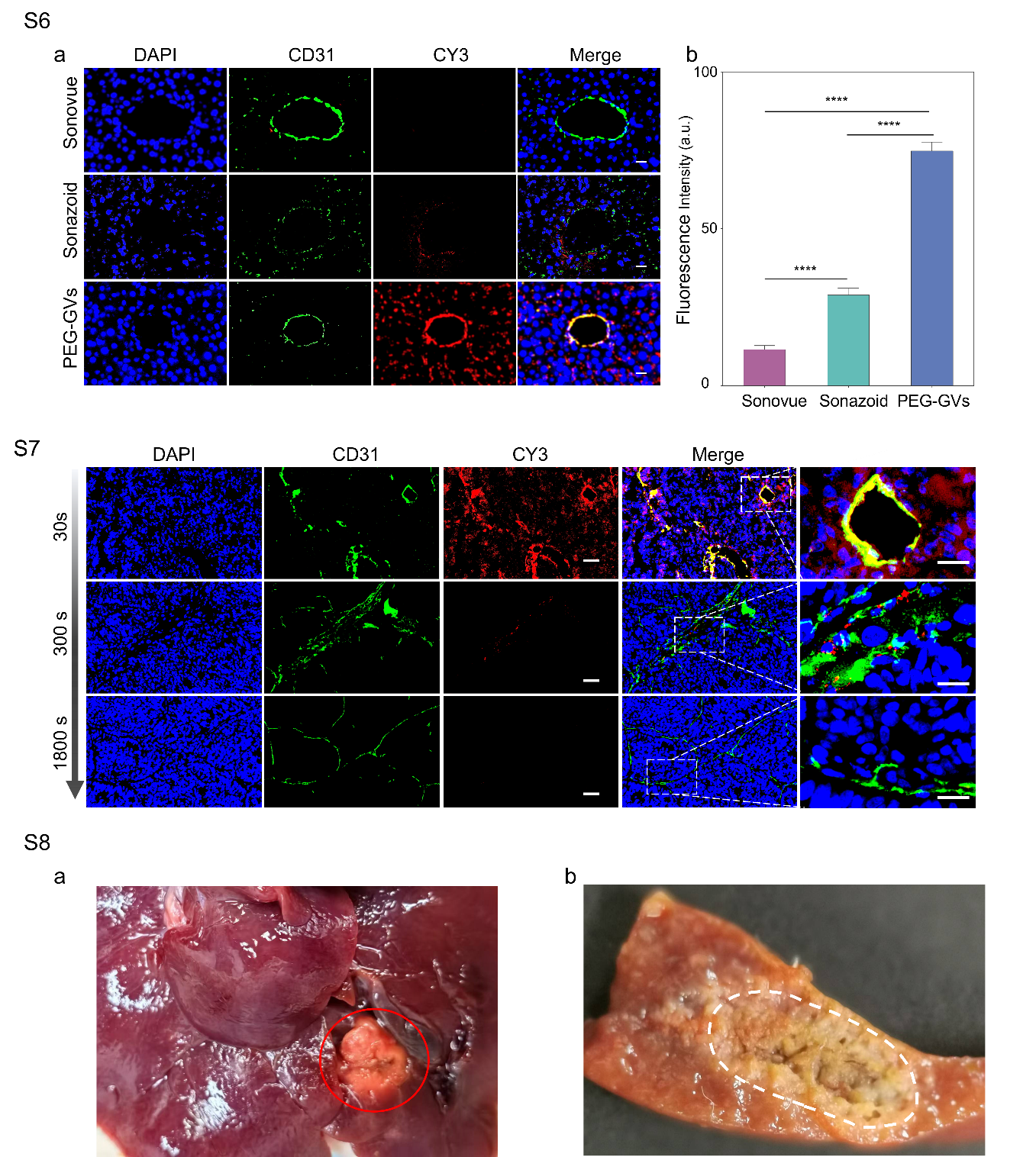


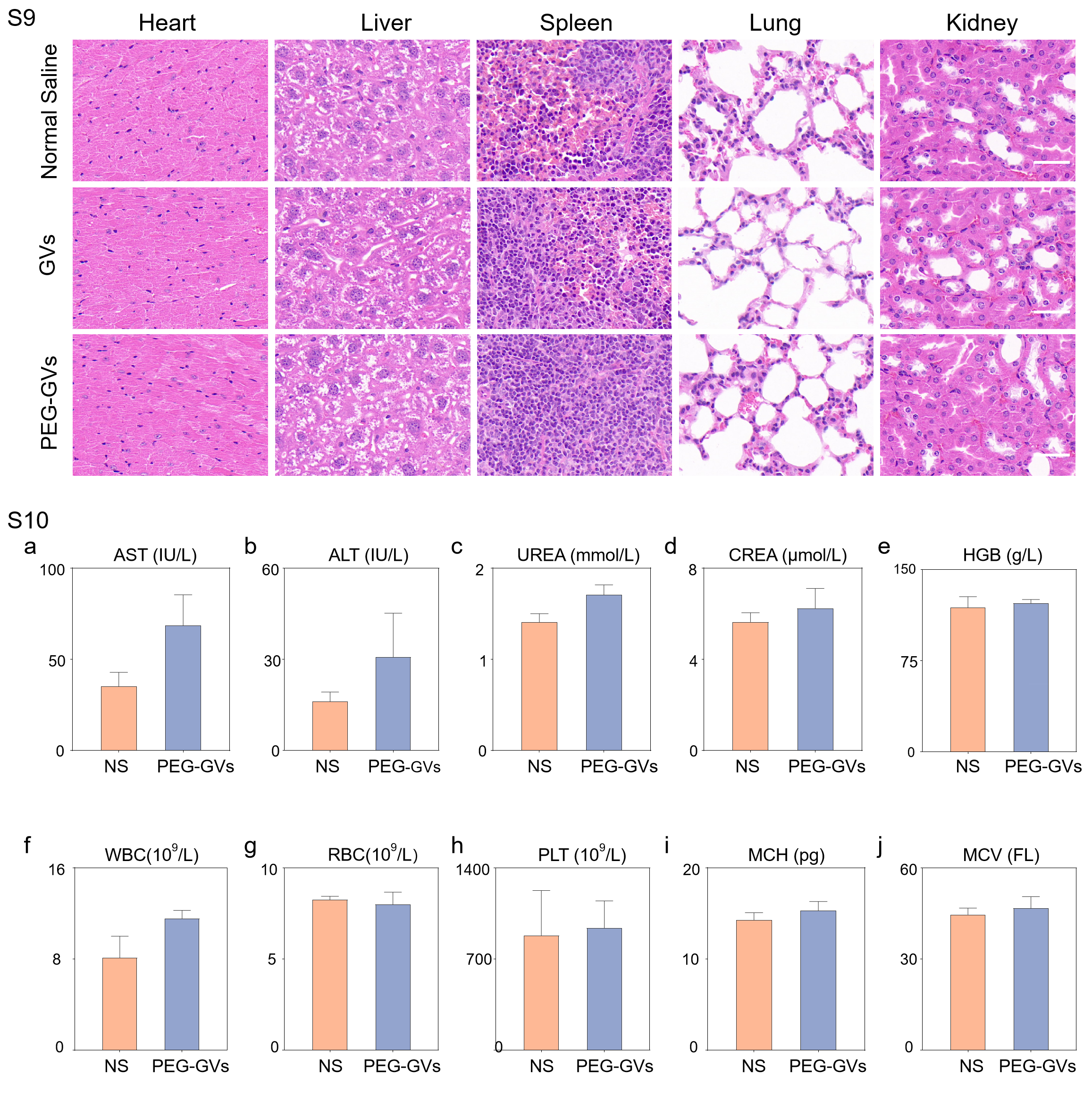


Supplemental Figures.

**S1**: Comparison of particle size of GVs and PEG-GVs. All samples were diluted in PBS solution at OD 500 = 0.1. **S2**: In vitro comparison of the stability of Sonovue, Sonazoid and PEG-GVs. With the extension of time, the state of Sonovue (SV), Sonazoid (SD) and PEG-GVs (PGVs) floated and destructed. These contrast agents were prepared as solutions and then placed at 4℃. **S3**: Wave spectrum quantification of the second harmonic imaging. **** p < 0.0001. **S4**: Comparison of the normalized area under the curve (AUC) within 900 s. **** p < 0.0001. **S5**: **a, b,** Distribution of contrast agents in the liver at 120 s **(a)** and 600 s **(b)** after injection, respectively. Scale bar: 1000 µm. **S6**: **a, b,** Intrahepatic fluorescence distribution in liver after injection of contrast agents (CY3-labeled PEG-GVs, DIO-labeled Sonazoid and Sonovue) 1 h **(a)** and the quantification **(b)**. Scale bar: 20 µm. **S7**: Distribution of CY-3-labeled PEG-GVs in liver tumors after injection of PEG-GVs at 30 s, 300 s, 1800 s. Scale bar: 20 µm. **S8**: **(a)** The VX2 in - situ liver carcinoma model was successfully established. **(b)** Macroscopic views of the liver specimens post - radiofrequency ablation revealed that the ablation area turned grayish white (white dotted circles). **S9**: Biosafety analysis of mice. Representative H&E sections of the main organs (heart, liver, spleen, lung, and kidney) from the mice received with normal saline, GVs, or PEG-GVs after 7 days. Scale bar: 50 µm. **S10**. **a-j**. Hematological detection of liver function **(a, b)**, kidney function **(c, d)**, and blood count **(e-j)** 3 days after intravenous injection of normal saline or PEG-GVs in mice.
